# Evidence of Evolving Extraintestinal Enteroaggregative *Escherichia coli* ST38 Clone

**DOI:** 10.3201/eid2011.131845

**Published:** 2014-11

**Authors:** Marie Anne Chattaway, Claire Jenkins, Holly Ciesielczuk, Martin Day, Vivienne DoNascimento, Michaela Day, Irene Rodríguez, Alieda van Essen-Zandbergen, Anne-Kathrin Schink, Guanghui Wu, John Threlfall, Martin J. Woodward, Nick Coldham, Kristina Kadlec, Stefan Schwarz, Cindy Dierikx, Beatriz Guerra, Reiner Helmuth, Dik Mevius, Neil Woodford, John Wain

**Affiliations:** Public Health England, Colindale, London, UK (M.A. Chattaway, C. Jenkins, H. Ciesielczuk, Martin Day, V. DoNascimento, Michaela Day, J. Threlfall, N. Woodford, J. Wain);; Hospital Universitario Ramón y Cajal, Madrid, Spain (I. Rodríguez);; University of East Anglia, Norfolk, UK (J. Wain); Federal Institute for Risk Assessment, Berlin, Germany (I. Rodríguez, B. Guerra, R. Helmuth);; Central Veterinary Institute of Wageningen, Lelystad, the Netherlands (A. van Essen-Zandbergen, C. Dierikx, D. Mevius);; Friedrich-Loeffler-Institut, Neustadt-Mariensee, Germany (A.-K. Schink, K. Kadlec, S. Schwarz);; Animal Health and Veterinary Laboratories Agency, Weybridge, UK (G. Wu, M.J. Woodward, N. Coldham)

**Keywords:** Escherichia coli, enteroaggregative, ESBL, extended-spectrum β-lactamase, extraintestinal, ST38, diarrheagenic E. coli, multiple drug resistance, Germany, the Netherlands, United Kingdom, bacteria

**To the Editor:** Several clones of extended-spectrum β-lactamase (ESBL)–producing extraintestinal pathogenic *Escherichia coli* (ExPEC) have globally expanded their distribution, including multilocus sequence types (MLSTs) ST38, ST131, ST405, and ST648 (*1*). ExPEC infections often originate from the patient’s own intestinal flora, although the degree of overlap between diarrheagenic *E. coli* and ExPEC pathotypes is unclear. Relatively little is known about antimicrobial drug resistance in the most common diarrheagenic *E. coli* groups, including enteroaggregative *E. coli* (EAEC), and bacterial gastroenteritis is generally managed without use of antimicrobial drugs.

The ability of diarrheagenic *E. coli* to cause extraintestinal infections has been shown in previous studies: a study among children in Nigeria linked EAEC to uropathogenic clonal group A (*2*), and a study in Brazil showed that EAEC markers were present in 7.1% of the *E. coli* isolates from urinary tract infections (*3*). Neither of these studies identified clonal lineages of EAEC specifically associated with extraintestinal infections.

We conducted this study to establish the presence and characteristics of ESBL-producing EAEC in a well-defined collection of ESBL-producing isolates (*4*). The isolates were from human and animal sources in Germany, the Netherlands, and the United Kingdom. The study was conducted at Public Health England during January–April 2013.

DNA from 359 ESBL isolates (*4*) was screened for the presence of the EAEC transport regulator gene (*aggR*), located on the EAEC plasmid, by using a real-time PCR assay and the following primers and probe: AggR_F 5′-CCATTTATCGCAATCAGATTAA-3′ AggR_R 5′-CAAGCATCTACTTTTGATATTCC-3′, AggR_P Cy5-CAGCGATACATTAAGACGCCTAAAGGA-BHQ. The amplification parameters were 50°C for 2 min, 95°C for 2 min, and 40 cycles at 95°C for 10 s and at 60°C for 20 s. Isolates positive for *aggR* were confirmed to be *E. coli* by using the Omnilog GenIII MicroPlate (Biolog, Hayward, CA, USA). Serotyping was done by using standard methods (*5*).

The phylogroup was determined for each isolate, and isolates were then assigned to 1 of the 4 major *E. coli* groups: A, B1, B2, and D (*6*). A microarray was used to detect ESBL genes, such as *bla*_CTX-M_, at the group level, as previously described (*4*). The antimicrobial drug susceptibilities of EAEC isolates were determined by using the agar incorporation method, as described in the British Society for Antimicrobial Chemotherapy guidelines (*7*).

Virulence factors associated with intestinal and extraintestinal infection (*8*) and with EAEC were investigated as previously described (*9*). We assigned a virulence score (total number of virulence factor genes detected; maximum possible score 22) and a resistance score (total number of drug classes; maximum score 11) to each isolate.

We isolated 11 EAEC from humans. Eight of the EAEC were isolated from urine specimens, and 1 was isolated from a blood culture; 63% belonged to phylogroup D (Table). EAEC ST38, the most common (55%) ST, was significantly associated with extraintestinal sites in the subset of 140 human isolates (Fisher exact test, p<0.0001).

**Table:** Characteristics of human-derived ESBL-producing enteroaggregative *Escherichia coli* isolates from sources in Germany, the Netherlands, and the United Kingdom*

Isolate	Serotype†	ST	Cplx‡	Country	Source	Phylotype	*aggR§*	Plasmidic ESBL	
ESBL-723	OR:H30	38	38	UK	Urine	D	+	CTX-M-15	
ESBL-746	O125ac:H30	38	38	UK	Urine	D	+	CTX-M-15	
ESBL-884	O19a:H30	38	38	UK	Urine	D	+	CTX-M-14	
ESBL-831	O19a:H30	38	38	UK	Urine	D	+	CTX-M-14	
ESBL-815	O19a:H30	38	38	UK	Blood	D	+	CTX-M-15	
ESBL-26	O153:H30	38	38	Netherlands	Urine	D	+	CTX-M-51	
ESBL-221	O92:H33	34	10	Germany	Feces	A	+	CTX-M-3	
ESBL-45	O?:H26	58	155	Netherlands	Urine	B1	+/−	CTX-M-14	
ESBL-46	O?:H-	694	None	Netherlands	Urine	A	+/−	CTX-M-15	
ESBL-48	O15:H1	545	None	Netherlands	Urine	D	+/−	CTX-M-1	
ESBL-64	O?:H23	224	None	Netherlands	Urine	B1	+/−	CTX-M-1	

*All isolates were collected in 2009 (*4*). ESBL, extended-spectrum β-lactamase. ST, sequence type. †H- not motile; O?, O unidentifiable; R, rough reaction. ‡Cplx–ST complex comprising single-locus variants. *§aggR*, enteroaggregative *E. coli* regulatory gene; +, positive in screen and isolates; −, negative in screen and isolates; +/−, positive in screen but negative in isolates, indicating unstable plasmid.

In this study, we identified multidrug-resistant EAEC isolates belonging to ST38; the isolates had various somatic antigens and *bla*_CTX-M_ genes (Table). The multiple somatic antigens, variety of antimicrobial drug–resistance scores, and variety of gene complements in this successful ST indicate multiple acquisitions of virulence markers, rather than clonal expansion from a single source (Table; Technical Appendix Figure).

In the MLST public database, which contained 5,143 *E. coli* entries in June 2013, ST38 is predominantly associated with urinary tract infections, but in-house MLST studies at the Gastrointestinal Bacteria Reference Unit, Public Health England, have shown that ST38 is a successful EAEC group. The presence of EAEC virulence factors, such as aggregative adherence fimbria AAFI and *aggR*, can mediate adherence of *E. coli* to bladder epithelial cells, but the virulence factors do not impart uropathogenic properties to all EAEC isolates (*10*). The ST38 strain described here probably originated from the gut and independently acquired the 2 phenotypes (uropathogenic *E. coli* [UPEC] and EAEC), which would suggest the emergence of a UPEC/EAEC hybrid strain. It seems likely that an ST38 *E. coli* strain adapted to EAEC plasmid carriage (a change that would help survival in the gut through increased adherence) has acquired UPEC virulence factors, facilitating the exploitation of an extraintestinal niche, the urinary tract.

Despite the characterization of numerous virulence factors, no single genetic feature currently defines EAEC or UPEC isolates. Because the EAEC ST38 strain had 4–7 ExPEC-associated virulence factors, we suggest that, on the basis of epidemiologic, microbiological, and molecular characteristics, the EAEC ST38 described in this study should be considered an ExPEC associated with uropathogenic infections. It is possible that the multidrug-resistant EAEC ExPEC group has expanded globally but is currently underreported. We therefore urge testing for the EAEC genotype in all clinical studies of *E. coli* pathotypes.

Our findings show the potential for EAEC, previously considered a gut pathogen, to cause extraintestinal infection. We suggest that the UPEC/EAEC pathotype may be an evolving clonal group. In particular, a single sequence type, ST38, was associated with multidrug resistance and with urinary tract infection in humans.

Technical Appendix Virulence factors and antimicrobial drug resistance gene content of enteroaggregative *Escherichia coli* isolates, grouped by phylogroup.

## References

[1] Pitout JD. Extraintestinal pathogenic *Escherichia coli*: a combination of virulence with antibiotic resistance. Front Microbiol. 2012;3:9. 10.3389/fmicb.2012.00009 PMC3261549 22294983

[2] Wallace-Gadsden F, Johnson JR, Wain J, Okeke IN. Enteroaggregative *Escherichia coli* related to uropathogenic clonal group A. Emerg Infect Dis. 2007;13:757–60. 10.3201/eid1305.061057 17553259 PMC2738470

[3] Abe CM, Salvador FA, Falsetti IN, Vieira MA, Blanco J, Blanco JE, Uropathogenic *Escherichia coli* (UPEC) strains may carry virulence properties of diarrhoeagenic *E. coli.* FEMS Immunol Med Microbiol. 2008;52:397–406. 10.1111/j.1574-695X.2008.00388.x 18336383

[4] Wu G, Day MJ, Mafura MT, Nunez-Garcia J, Fenner JJ, Sharma M, Comparative analysis of ESBL-positive *Escherichia coli* isolates from animals and humans from the UK, the Netherlands and Germany. PLoS ONE. 2013;8:e75392. 10.1371/journal.pone.0075392 24086522 PMC3784421

[5] Gross RJ, Rowe B. Serotyping of *Escherichia coli*. In: Sussman M, editor. The virulence of *Escherichia coli*. Cambridge (UK): Cambridge University Press; 1985. p. 345–60.

[6] Clermont O, Bonacorsi S, Bingen E. Rapid and simple determination of the *Escherichia coli* phylogenetic group. Appl Environ Microbiol. 2000;66:4555–8. 10.1128/AEM.66.10.4555-4558.2000 11010916 PMC92342

[7] Howe RA, Andrews JM. BSAC standardized disc susceptibility testing method (version 11). J Antimicrob Chemother. 2012;67:2783–4. 10.1093/jac/dks391 23095231

[8] Johnson JR, Stell AL. Extended virulence genotypes of *Escherichia coli* strains from patients with urosepsis in relation to phylogeny and host compromise. J Infect Dis. 2000;181:261–72. 10.1086/315217 10608775

[9] Jenkins C, Chart H, Willshaw GA, Cheasty T, Smith HR. Genotyping of enteroaggregative *Escherichia coli* and identification of target genes for the detection of both typical and atypical strains. Diagn Microbiol Infect Dis. 2006;55:13–9. 10.1016/j.diagmicrobio.2005.10.019 16500068

[10] Boll EJ, Struve C, Boisen N, Olesen B, Stahlhut SG, Krogfelt KA. Role of enteroaggregative *Escherichia coli* virulence factors in uropathogenesis. Infect Immun. 2013;81:1164–71. 10.1128/IAI.01376-12 23357383 PMC3639612

