# Supplementary material for: Evidence of Evolving Extraintestinal Enteroaggregative Escherichia coli ST38 Clone
Source: Emerg Infect Dis. 2014 Nov;20(11):1935–7. doi: 10.3201/eid2011.131845 (PMC4214294; doi:10.3201/eid2011.131845)
Supplement: Technical Appendix — Virulence factors and antimicrobial drug resistance gene content of enteroaggregative Escherichia coli isolates, grouped by phylogroup. [file 13-1845-Techapp-s1.pdf]

Article DOI: http://dx.doi.org/10.3201/eid2011.131845

Evidence of Evolving Extraintestinal Enteroaggregative *Escherichia coli* ST38 Clone

Technical Appendix

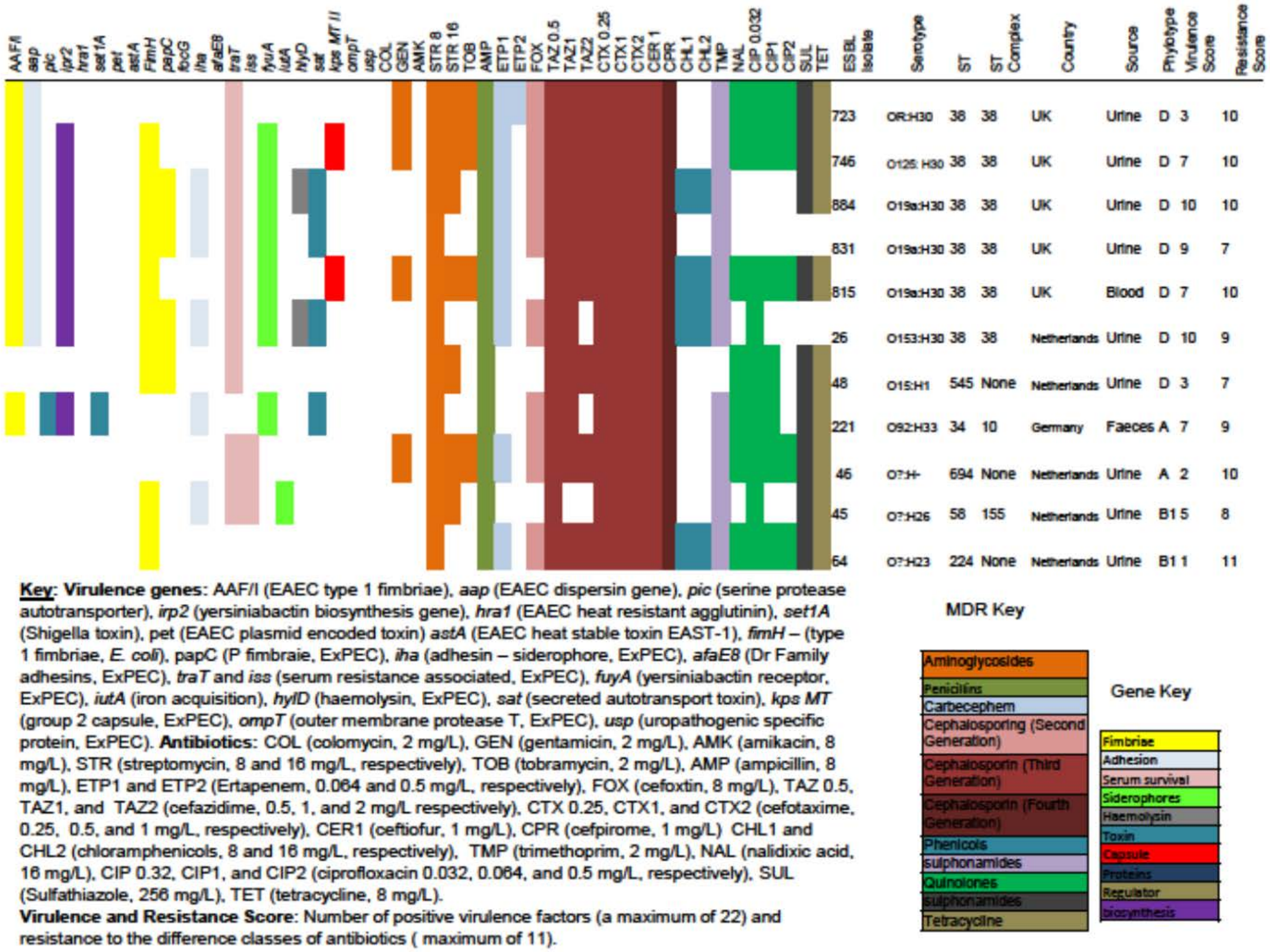

Technical Appendix Figure. Virulence factors and antimicrobial drug resistance gene content of Enteroaggregative *Escherichia coli* (EAEC) isolates, grouped by phylogroup.
